# SnoRNA and SNHG in Bladder Cancer: Molecular Mechanisms and Clinical Significance

**DOI:** 10.3390/cimb48070662

**Published:** 2026-06-27

**Authors:** Galiya Gimalova, Irina Gilyazova, Elza Khusnutdinova, Valentin Pavlov

**Affiliations:** Institute of Urology and Clinical Oncology, Department of Medical Genetics and Fundamental Medicine, Bashkir State Medical University, 450008 Ufa, Russia; gilyasova_irina@mail.ru (I.G.); ekhusnutdinova@bashgmu.ru (E.K.);

**Keywords:** snoRNA, *SNHG*, bladder cancer, SNORS, therapeutic targets

## Abstract

This review summarizes current data on the role of small nucleolar RNAs (snoRNAs) and their host genes (*SNHGs*) in the development of bladder cancer (BC). It examines snoRNA biogenesis, classical functions (rRNA modification), and non-canonical oncogenic mechanisms, including microRNA sponging, sdRNA production, and protein interactions (EZH2, DNMT3A, hnRNPK). The factors involved in the deregulation of snoRNA/*SNHG* expression during tumour transformation are described, such as amplifications, epigenetic changes, and transcriptional control (c-Myc, p53). Studies have shown that in BC, the majority of snoRNAs/*SNHGs* (*SNHG1*, *SNHG3*, *SNHG6*, *SNHG13*, *SCARNA12*) act as oncogenes, activating the PI3K/AKT, Wnt/β-catenin, NF-κB, and c-Myc pathways, thereby enhancing proliferation, EMT, invasion, and metastasis. Suppressor molecules (*SNHG2*/*GAS5*) are also discussed. The clinical potential of snoRNAs as prognostic signatures (SNORS), diagnostic biomarkers (SNHG1 in urine), and therapeutic targets (e.g., SNHG3) is analyzed. Thus, snoRNAs and *SNHGs* represent a promising class of molecules for the development of new diagnostic and therapeutic approaches for BC, although further investigation in prospective studies is required.

## 1. Introduction

Bladder cancer (BC) is one of the most common malignancies worldwide. Globally, more than 614,000 new cases of BC were diagnosed in 2022 [1]. However, standard diagnostic methods have limited sensitivity, and treatment for advanced forms remains insufficiently effective. Despite significant advances in diagnostics and treatment, the high risk of recurrence and progression, particularly in muscle-invasive forms, necessitates the identification of new molecular markers and therapeutic targets.

Recent research has focused on the role of non-coding RNAs in regulating gene expression during the development of malignancies. Although the majority of work in this area has traditionally concentrated on microRNAs and long non-coding RNAs, increasing evidence points to the importance of small nucleolar RNAs, one of the most abundant and evolutionarily conserved families of small non-coding RNAs.

Small nucleolar RNAs (snoRNAs) are a group of non-coding RNAs, 60–300 nucleotides in length, that form functional ribonucleoprotein complexes (snoRNPs). They are localized in the nucleolus and play a key role in the modification of ribosomal RNAs, which is necessary for their proper folding and assembly into ribosomes, as well as for the subsequent functioning of the entire translational apparatus. Increasing evidence is emerging regarding the involvement of snoRNAs in tumour formation and progression.

The aim of this review is to systematize data on snoRNA expression, mechanisms of function, clinical significance and therapeutic potential in BC.

## 2. Materials and Methods

The review strategy included a literature search in publicly available databases: PubMed (https://pubmed.ncbi.nlm.nih.gov), Web of Science, Scopus, NCBI (https://www.ncbi.nlm.nih.gov), Ensembl (https://www.ensembl.org), and specialized resources such as TCGA portal (https://www.cancer.gov/tcga). All resources were accessed on 11 May 2026. Full-text articles in English published primarily between 2015 and 2025 were analyzed, together with key earlier studies describing fundamental aspects of snoRNA biogenesis.

Search strategy details. The search was performed using the following Boolean string in PubMed: (“snoRNA” OR “*SNHG*” OR “SNORD” OR “SNORA”) AND (“bladder cancer” OR “urothelial carcinoma”) AND (“biomarker” OR “prognosis” OR “therapy” OR “mechanism”). Equivalent queries were adapted for Web of Science, Scopus, and other databases. After removing duplicates, titles and abstracts were screened for relevance. Full-text articles were assessed for eligibility based on the inclusion and exclusion criteria. The final selection comprised 48 articles, which form the reference list of this review. A detailed breakdown of the search and selection process is available from the authors upon request.

Inclusion criteria. Original studies and review articles were included if they: (1) described the expression of snoRNA or *SNHG* genes in bladder cancer; (2) examined the molecular mechanisms of their action (including miRNA sponging, sdRNA formation, and protein interactions); (3) analyzed the correlation with clinical parameters (stage, metastasis, survival). Studies examining the non-canonical functions of snoRNA in other malignancy types were also considered for comparative analysis.

Exclusion criteria. Articles focusing solely on other types of non-coding RNAs (microRNAs, long non-coding RNAs) without mentioning snoRNAs/*SNHGs*, as well as in silico studies without experimental validation, conference abstracts, and preprints, were excluded from the review.

Data analysis and synthesis. We paid particular attention to transcriptomic studies and signalling pathway analysis to identify regulatory mechanisms associated with bladder carcinogenesis. Information on molecular targets, signalling pathways and clinical significance was extracted from the selected publications. The data were organized in a summary table, which includes the snoRNA/*SNHG* type, oncogenic or tumour suppressor role, molecular mechanism, cellular effects, clinical associations and references. Additionally, key prognostic signatures (SNORS) and therapeutic targets are highlighted in a separate section.

## 3. Biogenesis and Functions of snoRNA

This class of noncoding RNAs is encoded by introns of non-coding and, to some extent, protein-coding genes, called snoRNA host genes (small nucleolar host genes, SNHG) [2]. A significant portion of snoRNAs are encoded by long non-coding RNA genes [3]. In these genes, lncRNA regions in the intronic regions are flanked by snoRNA sequences. The excised intron is not degraded; instead, snoRNAs are formed [4].

Furthermore, a number of snoRNA host genes are housekeeping genes required for ribosome biogenesis and function.

The pre-snoRNA processing required for the formation of the mature molecule occurs primarily in the nucleoplasm. This process involves specific nucleases (RNase III, 5′→3′ and 3′→5′ exonucleases), as well as proteins that subsequently participate in the stabilization and formation of small nucleolar ribonucleoproteins. Assembly of snoRNP complexes is necessary for the localisation of these RNAs in the nucleoli and for their functioning: snoRNAs form a scaffold for the assembly of protein complexes, direct specific recognition of target RNAs, and define the site of post-transcriptional modification [5].

Mature snoRNPs contain conserved core proteins: for C/D boxes, SNU13 (15.5K), NOP58, NOP56, and the catalytic subunit fibrillarin (FBL); for H/ACA boxes—dyskerin (DKC1), NHP2, NOP10, and GAR1.

There are two main groups of snoRNAs: C/D boxes directing site-specific methylation (SNORD) [6] and H/ACA boxes directing pseudouridylation (SNORA) [5]. A class of molecules containing both C/D and H/ACA boxes is also known. The former, in interaction with specific proteins, participates in the formation of ribonucleoproteins containing two types of conserved sequences—the C and D structures. This group of snoRNAs binds to complementary rRNA targets via their antisense elements and regulates their 2′-O-methylation.

The second group forms H- and ACA-box-containing ribonucleoproteins. These snoRNAs bind to target RNAs and modify uridine bases to pseudouridine [7]. Pseudouridylation, the most common modification, is a mechanism for maintaining the stability of different classes of RNA and also influences ribosome formation. Recent studies show that snoRNAs are also involved in ac4C modifications of rRNA [8]. Modifications produced by snoRNAs protect target RNAs from nuclease degradation and influence their conformation, promoting improved RNA-RNA and RNA–protein interactions [9].

A number of snoRNAs, forming specific protein complexes, participate in pre-rRNA processing: C/D box RNAs SNORD3A, SNORD8, SNORD14A/SNORD14B and SNORD22, and H/ACA box RNAs SNORA10, SNORA30, and probably also SNORA73/E1, E2 and E3 [3].

A small class of rare snoRNAs does not contain regions complementary to rRNAs, i.e., they are not involved in their classical post-transcriptional modifications [3]. Such snoRNAs may participate in the regulation of alternative splicing of pre-mRNA [10,11], direct the modification of other cellular RNAs, or act as RNA chaperones [12].

Some snoRNAs (e.g., SNORA3, SNORA71, SNORD113) are known to translocate from the nucleus to the cytoplasm in response to oxidative or metabolic stress, regulate RNA-protein interactions, and participate in the formation of stress granules. Moreover, disruption of this process, caused, for example, by increased snoRNA expression resulting from chronic stress, can contribute to the formation of stable stress granules, protein aggregation and, consequently, play a role in the development of oncological and neurodegenerative diseases [13].

**Figure 1:**
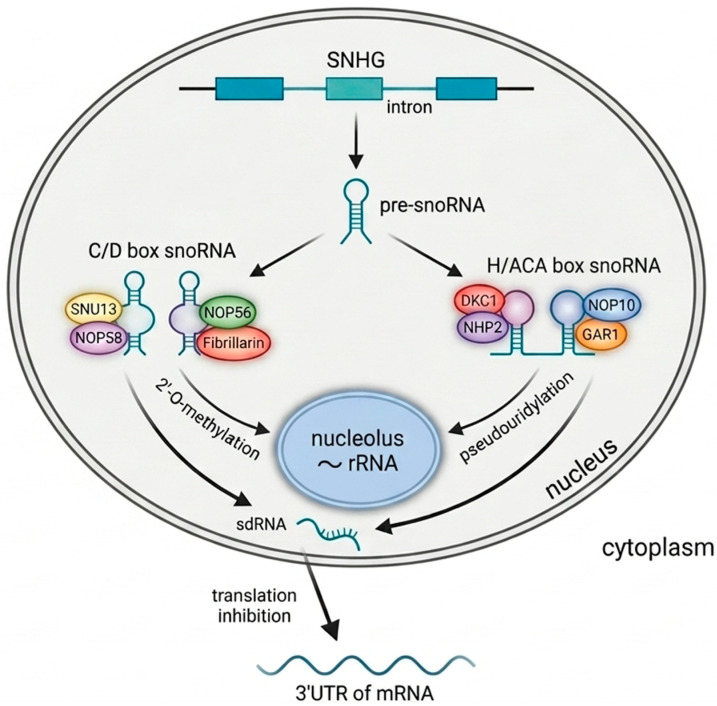
General scheme of snoRNA biogenesis and functions.

The development of high-throughput sequencing technologies has made it possible to detect even smaller RNA molecules in cells—snoRNA-derived RNAs (sdRNAs). Furthermore, the formation of these fragments has been shown not to be random but rather a regulated cellular mechanism. SdRNAs, like microRNAs, regulate translation or form complexes with proteins such as hnRNPs, and influence splicing, chromatin accessibility, and mitochondrial function [9]. The main steps of snoRNA biogenesis, assembly into C/D and H/ACA snoRNPs, classical rRNA modifications, and the non-canonical sdRNA pathway are summarized in Figure 1. Thus, the range of snoRNA functions extends far beyond their classical role. Next, we will consider how deregulation of these processes contributes to malignant transformation.

## 4. The Role of snoRNA in Oncogenesis

The role of snoRNA in tumour development, including BC, is associated with increased proliferation, invasion, and resistance to therapy via oncogenic pathways.

In tumour development, increased expression of snoRNA host genes (e.g., *SNHG1*, *SNHG3*, *SNHG7*, *SNHG20*) is often observed. Causes of this phenomenon include amplification of these genes [14], as well as mutations and CNVs [15,16,17]. Epigenetic deregulation factors such as promoter hypomethylation and histone acetylation (H3K27ac) of these genes also play a significant role in oncogenesis.

Furthermore, snoRNA levels are controlled by transcription factors. In particular, overexpression of the c-Myc oncogene leads to increased transcription, while abnormal activation of the *EZH2* gene promotes promoter hypermethylation (H3K27me3) and leads to transcriptional repression of snoRNA genes [7,18]. Increased transcription is also facilitated by the loss of activity of tumour suppressors, such as p53, leading to suppression of apoptosis. A well-known mechanism for tumour cell survival and maintenance of high metabolic activity even under stress is post-transcriptional stabilization through interaction with proteins such as hnRNPK and NOP58, as well as a number of regulatory RNAs, including snoRNA [7]. The role of this mechanism is to maintain the stability of oncogene mRNAs against the action of various endonucleases, protect against the degradation of anti-apoptotic factors, and stabilize RNAs responsible for EMT.

### 4.1. Molecular Mechanisms of the Oncogenic Action of snoRNA

We have already discussed that the main classical function of snoRNA is considered to be the modification of rRNA. In the context of carcinogenesis, however, the non-canonical functions of these RNAs are of greatest interest.

The host genes of snoRNAs predominantly exhibit oncogenic activity. Their increased expression leads to the activation of the PI3K/AKT, Wnt/β-catenin, NF-κB, and c-Myc pathways, which in turn promotes proliferation, EMT, invasion, and metastasis [7,18].

By stimulating the translation of oncogenes, they activate ribosome biogenesis, which is critical for the uncontrolled proliferation of tumour cells. SNHGs often act through sdRNA (sno-derived miRNA-like) and, similar to miRNAs, bind the 3′UTR of their target. These sdRNAs have been reported to suppress tumour suppressors or activate pro-oncogenic pathways in cancer [9,19].

It is also known that *SNHG* genes, functioning as competitive endogenous RNAs, can sponge suppressor microRNAs, activating EMT and angiogenesis [19,20].

**Figure 2:**
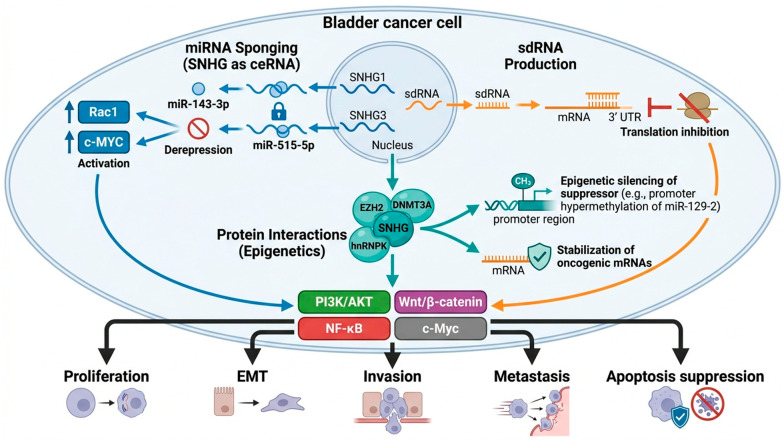
Main oncogenic mechanisms of snoRNA/SNHG in bladder cancer. The diagram illustrates three key oncogenic cascades: miRNA sponging (SNHG as ceRNA), sdRNA production, and protein interactions. These converge on PI3K/AKT, Wnt/β-catenin, NF-κB, and c-Myc pathways, leading to proliferation, EMT, invasion, metastasis, and apoptosis suppression. Arrow colors: blue—miRNA sponging; orange—sdRNA; green—protein interactions.

SnoRNAs also stabilize oncomiRNP targets, preventing their premature degradation and promoting their excessive accumulation. These targets include the oncogenic protein EIF4A3, which is involved in splicing and the regulation of protein synthesis, and laminin LAMC2, the excess of which helps tumours invade healthy tissue and metastasise [7,18]. Figure 2 illustrates the three main oncogenic mechanisms—miRNA sponging, sdRNA production, and protein interactions—converging on PI3K/AKT, Wnt/β-catenin, NF-κB, and c-Myc pathways, ultimately leading to proliferation, EMT, invasion, metastasis, and apoptosis suppression. The mechanisms described above are universal across many types of cancer. Next, we will analyze specific data obtained in BC.

### 4.2. The Role of snoRNA in the Development of BC

It should be noted that most snoRNAs and the genes encoding them act as oncogenes, enhancing proliferation, invasion, and EMT, while only a few act as suppressors, inhibiting these processes [18,21]. In the following sections, we distinguish findings that have been directly demonstrated in BC from those that are based on studies in other cancer types (e.g., colorectal, breast, lung, pancreatic, prostate). For mechanisms that have not yet been validated in BC, we explicitly state that further investigation is needed.

As already mentioned, during the development of malignant neoplasms, including BC, activation of the *SNHG1*, *SNHG3*, *SNHG7*, and *SNHG20* genes is observed. They act through the sponging of microRNAs or the activation of the Wnt/β-catenin and c-MYC pathways. Moreover, these processes correlate with the tumour stage, the extent of the process, tumour metastasis to lymph nodes, and disease prognosis.

Among the most well-studied is the *SNHG1* gene (small nucleolar RNA host gene 1), which encodes an lncRNA. This gene generates eight C/D box snoRNAs through alternative splicing: SNORD22 (involved in 18S rRNA maturation), SNORD25 (involved in 18S rRNA methylation), SNORD26, SNORD27, SNORD28, SNORD29, SNORD30, SNORD31 (involved in 28S rRNA methylation). Its increased expression has been shown in many types of cancer, including BC [22,23,24]. Among the described mechanisms, several have been directly validated in BC (e.g., SNHG1/miR-143-3p/EZH2, SNHG1/DNMT3A/miR-129-2/Rac1), whereas others, such as the SNHG1/miR-199a-3p/TFAM axis, have been demonstrated primarily in breast and prostate cancer and require BC-specific confirmation.

It is also known that increased *SNHG1* expression in BC is associated with a poor prognosis. In vivo experiments showed that downregulation of SNHG1 expression inhibited tumour growth and metastasis of BC cells [25]. SNHG1 has been shown to exert its oncogenic effects through increased tumour proliferation, partly through activation of the PI3K/AKT signalling pathway and through regulation of the Wnt/β-catenin signalling pathway [26]. SNHG1 is also known to suppress apoptosis. Interestingly, SNORD28 can be further processed into short fragments that function as microRNAs, designated sno-miR-28. These microRNAs affect p53 protein stability, and high SNORD28 levels in BC correlate with increased tumour cell proliferation [27]. Another interesting snoRNA from this list is SNORD22. Its overexpression has been shown to be associated with EMT and metastasis. Furthermore, it has been shown that SNHG1 can function as a competitive endogenous RNA and lead to a decrease in *MDM2* expression by sponging microRNA-9-3p. In turn, MDM2 stimulates ubiquitination and degradation of the transcription factor PPARγ, which is also associated with cancer cells [22]. In the cytoplasm of cancer cells, SNHG1 can also sponge microRNA-143-3p. In the nucleus, SNHG1 can interact with the EZH2 methyltransferase and regulate histone methylation in the promoter of the CDH1 cadherin gene, which mediates cell–cell adhesion in epithelial tissues. This ultimately alters the biological behaviour of BC cells [25]. Another study showed that SNHG1 binds to the DNMT3A methyltransferase and attaches it to the miR-129-2 promoter, causing hypermethylation and transcriptional suppression of this microRNA. Reduced miR-129-2 binding to Rac1 mRNA, in turn, leads to its stabilization and increased Rac1 protein levels, stimulating cell invasion in muscle-invasive BC. Increased Rac1 expression is associated with poor survival in patients with invasive BC [25,28].

Various studies, including TCGA data, suggest that high *SNHG1* gene expression is associated with more aggressive muscle-invasive bladder cancer (MIBC) and lower patient survival. *SNHG1* gene products are of interest as potential biomarkers and candidates for liquid biopsy, as elevated levels of SNHG1 and related SNORDs are detected even in the urine of BC patients [25,29]. While these findings are promising, most functional studies used a limited number of cell lines and xenografts, and the clinical associations are derived from retrospective TCGA analysis. Independent prospective validation is therefore needed.

Another snoRNA host gene with known oncogenic potential is *SNHG3*. It encodes SNORA73A and SNORA73B, which belong to the box H/ACA class [30]. Increased gene expression has been reported in various types of cancer and is associated with an unfavourable prognosis [31]. This is associated, among other things, with increased activity of the translational apparatus of tumour cells and a greater demand for snoRNA molecules. Suppression of *SNHG3* expression was found to inhibit proliferation, migration, invasion, and epithelial–mesenchymal transition of BC cells both in vitro and in vivo. It has been shown that SNHG3 positively regulates *GINS2*, a key participant in the DNA replication process that is overexpressed in many types of cancer by binding to microRNA-515-5p via a competing endogenous RNA mechanism. In addition, SNHG3 stabilizes *BMI1* gene mRNA and activates transcription factors, resulting in significant upregulation of the c-MYC and BMI1 pathways, followed by downregulation of cell–cell contact proteins (E-cadherin) and upregulation of motility proteins (N-cadherin, vimentin) [32]. This leads to increased EMT [7,21]. It should be noted that microRNA-515-5p in BC acts as an important tumour suppressor by downregulating the syndecan *SDC1* gene, which improves cell adhesion and reduces invasive capacity [33]. In turn, suppression of SNHG3 activity promotes increased expression of microRNA-515-5p and decreased expression of *GINS2* [31]. Functional validation of SNHG3 in BC was performed using several cell lines and xenograft models, with consistent results across two independent studies [31,32]. However, the patient cohorts used for clinical correlations were relatively small (approximately 80–100 cases per study), and larger prospective studies are warranted to confirm their prognostic value.

The *SNHG6* gene is also overexpressed in various cancer types, including colorectal cancer, hepatocellular carcinoma, lung cancer and BC. It encodes the snoRNA SNORD87. It is considered a potential biomarker of cancer aggressiveness and progression, as well as a marker of chemotherapy resistance [34]. At the cellular level, SNHG6 functions through cell cycle regulation and inhibition of apoptosis, and has also been implicated in EMT and increased cell migration and invasiveness in BC cells [34]. There is evidence that SNHG6 can also sponge miR-26a-5p, resulting in decreased levels of the latter. Normally, miR-26a-5p suppresses the MAPK6 pathway, thus acting as a tumour suppressor [35]. The mechanisms involving miR-125b and miR-26a-5p have been validated in BC cells. However, the proposed role of SNHG6 in chemotherapy resistance [34] was primarily demonstrated in other tumour types and awaits BC-specific confirmation.

The *SNHG5* gene encodes two snoRNAs with tumour suppressor activity—SNORD50A and SNORD50B. They bind to the k-Ras protein and block its activation, suppressing the Ras-ERK signalling pathway responsible for tumour cell survival and migration [36]. Loss of these RNAs contributes to k-Ras hyperactivation and is associated with tumour aggressiveness. Low levels of SNORD50A/B usually correlate with poor patient survival. It should be noted that a number of studies have demonstrated the dual action of these RNAs: suppressive in the presence of mutated p53 and oncogenic in the presence of wild-type p53. In the latter case, they can promote p53 degradation, thereby supporting tumour survival [37]. Furthermore, there is evidence that SNHG5 suppresses the p27 protein, which blocks cell division, leading to increased proliferation, cell cycle activation and inhibition of apoptosis [38]. It is also known that lncRNA encoded by the *SNHG5* gene can sponge microRNA-26a-5p and activate its target, protein kinase GSK3β, which plays an oncogenic role in some cancers, including BC. This discrepancy in the effects of the products of the same gene (oncogenic lncRNA and suppressor snoRNA) can be explained by several factors: on the one hand, by localization peculiarities (failures in the transport of snoRNA from the nucleus to block k-Ras), and on the other hand, by extremely high levels of k-Ras in the tumour cell and a lack of snoRNA to bind it [39]. It should be noted that the dual role of SNHG5/SNORD50A/B, depending on p53 status, has been characterized primarily in breast and other cancers. In BC, the evidence is limited to bioinformatics analysis of TCGA data and a small number of functional experiments; therefore, the context-dependent functions of this locus in BC require further investigation. This example illustrates that the functions of snoRNAs and *SNHGs* are not unidimensional; similar context-dependent effects may exist for other molecules in this review (e.g., *SNHG1* has been reported to act as both an oncogene and a tumour suppressor depending on the cellular context). These complexities are summarized in Table 1 (see ‘Type’ column) and should be taken into account when interpreting functional studies.

The *SNHG13* (*DANCR*) gene encodes the snoRNA SNORD13. Elevated levels of SNORD13 stimulate the translation of oncoproteins and also influence EMT by activating genes associated with cell migration. Furthermore, acting as a ceRNA, SNHG13 sponges the suppressor microRNAs miR-149, miR-335 and miR-145. Functional studies have also shown that SNHG13 stimulates the migration, invasion and proliferation of cancer cells in vitro and enhances tumour growth and metastasis to lymph nodes in vivo [40,41]. Notably, the functional studies cited here were performed directly in BC cell lines and xenografts, providing BC-specific evidence for the oncogenic role of *DANCR*.

There is some evidence of the oncogenicity of several other snoRNA host genes in cancer, including *SNHG7* [42] and *SNHG12* [43]. *SNHG14-16* have also been identified, but the data are currently limited and require further validation in independent cohorts.

One of the few snoRNA host genes with a described suppressor role in the development of BC is *SNHG2*, also known as *GAS-5* (growth arrest-specific transcript 5). The expression level of this gene has been shown to be reduced in BC, and this level correlates significantly with clinical characteristics and prognosis. Increased expression of this gene stimulates apoptosis and suppresses bladder cell proliferation. It has been shown to function through interaction with the transcription factor *E2F4*, which leads to the suppression of transcription of the EZH2 oncogene [44].

Direct data on the role of individual snoRNAs in the development of BC are still limited, and they are most often studied in the context of the aforementioned host genes or fragments—sdRNA [18]. However, there are results from studies of snoRNA signatures. Small nucleolar RNA signatures (SNORS) are specific expression profiles of small nucleolar RNAs obtained through statistical selection of molecules based on their expression and association with survival or relapse. SNORS profile analysis allows highly accurate prediction of disease progression and patient survival, and can also be used as a biomarker for diagnosis and subtyping of various cancer types. Current data show that molecular scoring based on snoRNA signatures (including snoRNP-associated profiles) predicts overall survival and disease-free intervals for patients much more accurately than classical clinical assessment based on parameters such as tumour size, extent and metastasis (TNM).

Oncogenic snoRNAs are characterized by their elevated levels in cancer, a correlation with adverse clinical outcomes, and involvement in EMT activation, the PI3K-AKT signalling pathway, extracellular matrix (ECM) remodelling, and ribosome biogenesis [20].

In cancer, snoRNA dysregulation is observed primarily through CNVs and methylation. For example, the expression levels of SNORD19B and SNORD3 were directly related to their copy number. Furthermore, the expression level of U49A is directly related to the degree of methylation of its sites, while an inverse relationship is observed for SNORD3 and SNORD19B [20]. The TCGA-BLCA study identified the signature of five snoRNAs: SNORD113-9, SNORD3, SNORD49A, SNORD114-1, and SNORD19B [29]. The first four were found to have an oncogenic effect, while the last of these functions as a tumour suppressor. Below, we briefly review the function of each of these snoRNAs. Increased expression of SNORD113-9 correlates with activation of the PI3K-AKT signalling pathway and interaction with ECM components. This promotes EMT and tumour cell invasion and is associated with decreased overall patient survival. Another snoRNA, SNORD3 (also known as U3), promotes focal adhesion formation and inhibits apoptosis by downregulating the pro-apoptotic proteins Bak and CD20, which is associated with decreased progression-free survival. SNORD49A (U49A) modulates proteoglycan metabolism in cancer cells, accelerating the cell cycle, and its high expression correlates with decreased disease-specific survival. SNORD114-1 activates ErbB signalling pathways and extracellular matrix receptors, which significantly enhances the invasive potential of tumour cells. The independent function of SNORD19B remains to be elucidated.

In addition to those described above, several individual snoRNAs are known to play a role in the development of cancer. In particular, SNORD78 has been shown to increase the expression of the FSCN1 protein, which is associated with cell motility and mobility, thereby contributing to the progression of non-muscle-invasive bladder cancer (NMIBC). SNORD33 induces hypomethylation of ribosomal RNA (rRNA), stimulating ribosomogenesis, and thus actively promotes oncogenesis [18].

SCARNA12 (small cajal body-specific RNA 12) also exhibits oncogenic activity in BC. Its increased expression has been demonstrated in BC, suggesting that this RNA may play a role in signalling pathways associated with cell adhesion and the extracellular matrix. The interaction of SCARNA12 with the transcription factor H2AFZ plays a significant role in the formation and progression of cancer cells. This interaction leads to transcriptional modulation and activation of oncogenic signalling pathways, in particular, modulation of extracellular matrix signalling pathways, which is critical for cancer cell motility. Experiments with cell lines have shown that, conversely, silencing of SCARNA12 suppresses proliferation, migration and the ability of cells to invade. The oncogenic role of SCARNA12 has been validated in BC cell lines and xenografts, and the study by Lu et al. included a patient cohort of moderate size (*n* = 120). Nevertheless, independent replication is still lacking, and further studies are needed to establish its clinical utility [45].

It should also be noted that proteins that form ribonucleoprotein complexes with snoRNA are also of interest as potential biomarkers. For example, levels of the proteins fibrillarin (FBL) and dyskerin (DKC1) are often elevated in certain types of cancer [46,47].

Overall, the evidence presented in this review varies in strength. For *SNHG1*, *SNHG3*, and *SCARNA12*, functional studies in BC cell lines and xenografts are available, and clinical associations have been derived from TCGA or moderately sized cohorts. However, many mechanisms are extrapolated from other cancer types (e.g., colorectal, breast, lung), and direct validation in BC remains an open area for investigation. Reproducibility across independent cohorts and experimental models is a key limitation that should be addressed in future research.

Table 1 summarizes the results of studies illustrating both general patterns and unique features of individual snoRNAs and their genes in cancer.

**Table 1:** The role of snoRNA and *SNHG* in cancer development.

snoRNA/SNHG	Type (Oncogene/Suppressor)	Molecular Mechanism	Effect in BC Cells	Clinical Association	Key Experimental Evidence (Models/Sample Size)	References
*SNHG1*	Dual (oncogene, suppressor)	(1) Sponging miR-143-3p → derepression of EZH2. (2) Recruitment of DNMT3A to miR-129-2 promoter → hypermethylation → decreased miR-129-5p → increased Rac1. (3) Binding of EZH2 → H3K27me3 on CDH1 promoter → decreased E-cadherin. (4) SNORD28 (product of SNHG1) → sno-miR-28 → destabilization of p53. (5) SNORD22 → association with EMT/metastasis.	Increased proliferation, migration, invasion, EMT; suppression of apoptosis; enhanced stemness (Rac1)	Correlation with stage, lymph node status, metastases, poor survival (especially MIBC). Detected in urine—candidate for liquid biopsy.	BC cell lines (T24, EJ, 5637), xenograft, TCGA (~400 BC samples); urine samples (*n* = ~100)	[22,25,28,29]
*SNHG3*	Oncogene	(1) Sponging miR-515-5p → upregulation of GINS2. (2) Stabilization of BMI1 mRNA → activation of c-MYC/BMI1. (3) Downregulation of E-cadherin, ↑ N-cadherin, vimentin.	Increased proliferation, migration, invasion, EMT (in vitro and in vivo)	Association with poor prognosis, high stage.	BC cell lines (multiple), xenograft; patient cohorts (*n* ≈ 80–100 per study); TCGA	[18,30,31,32]
*SNHG5* (encodes SNORD50A/B)	Dual (suppressor with mutant p53; oncogenic with WT p53)	(1) SNORD50A/B bind to k-Ras → blocking of Ras-ERK (suppression). (2) With WT p53—promote p53 degradation. (3) Sponging miR-26a-5p → upregulation of GSK3β. (4) Suppression of p27 → cell cycle activation.	(1) With mutant p53: decreased proliferation, migration. (2) With WT p53: increased proliferation, suppression of apoptosis.	Low SNORD50A/B levels associated with aggressiveness and poor survival; however, role depends on p53 status.	BC cell lines (limited), TCGA analysis; primary evidence from other cancers (breast, colon)	[36,37,38,39]
*SNHG6*	Oncogene	(1) Sponging miR-125b → activation of Snail1/2 → EMT. (2) Sponging miR-26a-5p → derepression of MAPK6. (3) Encodes SNORD87.	Enhanced migration, invasiveness, EMT; cell cycle regulation, suppression of apoptosis	Potential biomarker of aggressiveness and chemoresistance.	BC cell lines, TCGA; chemoresistance data from other cancers	[34,35]
*SNHG7*	Oncogene	Sponging miR-2682-5p → activation of Wnt/β-catenin.	Increased proliferation, invasion, migration	Associated with poor prognosis.	BC cell lines (single study), TCGA	[40]
*SNHG12*	Potentially oncogenic (requires validation)	Requires further study	Stimulation of proliferation, invasion (preliminary data)	No data	Preliminary data, mostly bioinformatics; requires validation	[43]
*SNHG13* (DANCR)	Oncogene	(1) Sponging miR-149, miR-335, miR-145. (2) Activation of IL-11. (3) Encodes SNORD13.	Increased proliferation, migration, invasion (in vitro); enhances tumour growth and lymph node metastasis (in vivo)	Correlation with high stage, grade, metastasis.	BC cell lines, xenograft, lymph node metastasis model; patient correlation (n not specified)	[40,41]
*SNHG20*	Oncogene (general data; for BC, presumably)	Activation of Wnt/β-catenin (known from other cancers).	Increased proliferation, invasion; suppression of apoptosis	No direct data in BC.	No direct BC data; evidence from other cancers (colorectal, etc.)	[17]
*SNHG2* (GAS5)	Suppressor	Interaction with transcription factor E2F4 → suppression of EZH2 transcription.	Decreased proliferation; increased apoptosis	Expression reduced in BC; correlation with clinical characteristics and prognosis.	BC cell lines, clinical samples (*n* = ~80); TCGA	[44]
SCARNA12	Oncogene	Interaction with H2AFZ → modulation of ECM/adhesion gene transcription.	Increased proliferation, migration, invasion; knockdown inhibits growth and induces apoptosis	Elevated in BC; promising target.	BC cell lines (RT4, T24), xenograft; patient cohort (*n* = 120)	[45]
SNORD113-9	Oncogene (within SNORS)	Activation of PI3K-AKT, interaction with ECM components	Stimulation of EMT, invasion	Part of prognostic SNORS; associated with decreased overall survival.	TCGA-BLCA analysis (bioinformatics), no functional validation	[29]
SNORD3 (U3)	Oncogene (within SNORS)	Suppression of proapoptotic proteins Bak and CD20; involvement in focal adhesion	Suppression of apoptosis	Associated with decreased progression-free survival.	TCGA-BLCA analysis, functional data from other cancers	[29]
SNORD49A (U49A)	Oncogene (within SNORS)	Modulation of proteoglycan metabolism; acceleration of cell cycle	Stimulation of proliferation	Correlates with decreased disease-specific survival	TCGA-BLCA analysis, methylation studies	[29]
SNORD114-1	Oncogene (within SNORS)	Activation of ErbB signalling and ECM receptors	Stimulation of invasion	Associated with aggressive phenotype	TCGA-BLCA analysis	[29]
SNORD19B	Suppressor (within SNORS)	Mechanisms not studied	Presumably inhibits invasion	Part of SNORS.	TCGA-BLCA analysis	[29]
SNORD78	Oncogene	Upregulation of FSCN1 (protein associated with cell motility)	Increased cell motility, progression of NMIBC	Associated with progression of non-muscle-invasive BC.	BC cell lines (NMIBC models), TCGA	[18]
SNORD33	Oncogene	Induction of rRNA hypomethylation → stimulation of ribosome biogenesis	Stimulates oncogenesis (general growth promotion)	No data	In silico, TCGA; functional studies in other cancers	[18]
SNORD22 (product of *SNHG1*)	Oncogene	Involvement in EMT and metastasis	Stimulates invasion, metastasis	Correlation with BC aggressiveness (within SNHG1).	BC cell lines (within *SNHG1* studies), TCGA	[22]
SNORD28 (product of *SNHG1*)	Oncogene	Processing into sno-miR-28 → destabilization of p53	Increased proliferation		BC cell lines, p53 interaction studies	[27]

## 5. Clinical Significance of snoRNA in Cancer

**Figure 3:**
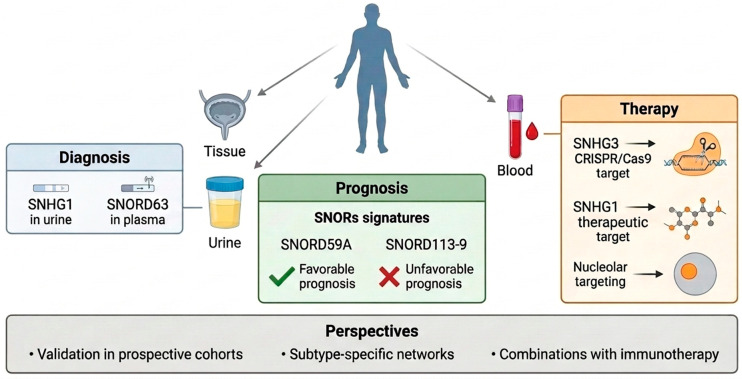
Clinical potential of snoRNAs in bladder cancer (diagnostic, prognostic, and therapeutic applications). All therapeutic strategies shown are at the preclinical stage and require further validation before clinical application.

As mentioned above, snoRNA and transcripts from *SNHG* host genes have become a popular research target in recent years and are considered a promising class of molecular markers in BC. The clinical potential of snoRNAs in bladder cancer, including diagnostic biomarkers, prognostic SNORS, and therapeutic targets, is summarized in Figure 3.

Their clinical significance stems from the fact that changes in the expression of this class of RNA reflect tumour properties such as aggressiveness and the potential for invasion and metastasis, and are associated with overall and disease-free survival. However, it is important to emphasize that the majority of evidence comes from retrospective cohorts and public databases (e.g., TCGA), and prospective validation is required before these markers can enter routine clinical practice.

The most studied prognostic signature associated with patient outcomes in BC is SNORS, which includes SNORD113-9, SNORD3, SNORD49A, SNORD114-1, and SNORD19B. Some molecules in this signature are associated with an unfavourable prognosis (SNORD113-9, SNORD3, SNORD49A, SNORD114-1), while SNORD19B is associated with a more favourable course of the disease. This SNORS has been shown to be associated not only with survival, but also with biological processes underlying tumour progression, including EMT, interactions with the extracellular matrix, focal adhesion, and PI3K-AKT and TGF-β signalling [20]. Despite this promising association, the SNORS has not yet been validated in independent prospective cohorts, and its performance relative to existing clinical models (e.g., based on TNM stage, grade) remains to be determined.

One candidate with diagnostic and prognostic potential is SNHG1. Studies have shown that SNHG1 is elevated in BC tissues and cells, and its elevated levels are associated with TNM staging, lymph node invasion, metastases, and relapse-free survival. Suppression of this RNA transcript reduces tumour cell proliferation, migration, and invasion in preclinical models [25]. However, these data are derived from a limited number of cell lines and xenografts, and clinical studies have been retrospective. Prospective evaluation is needed.

Another important candidate is SNHG3. Its levels are also elevated in BC. SNHG3 promotes proliferation, migration, invasion, and EMT. In the clinical context, this molecule is considered a potential marker of aggressive tumour progression, as well as a possible prognostic marker [31]. Nevertheless, these clinical associations are derived from retrospective analyses of relatively small cohorts, and prospective validation is required before SNHG3 can be considered a clinically reliable marker.

Beyond the specific candidates discussed above, several general challenges apply to the entire field of snoRNA-based biomarkers. Despite the promise of snoRNA-based biomarkers, several significant hurdles must be overcome before clinical translation. Most published studies are retrospective, often using convenience sample sets or public databases (e.g., TCGA), and independent prospective cohort studies are urgently needed. There is currently no standardized protocol for snoRNA detection; methods range from qPCR to RNA-seq, and normalization strategies differ. The choice of sample type (tissue, urine, or plasma) influences detection sensitivity and specificity, and pre-analytical variables are rarely addressed. Clinically meaningful cutoff values have not been established, and the performance of snoRNA markers has not been systematically compared with existing clinical tools such as urine cytology, cystoscopy, or FDA-approved urinary biomarkers. Moreover, the utility of these markers may differ between NMIBC and MIBC and across molecular subtypes. Addressing these challenges is essential for eventual clinical implementation.

In addition to their diagnostic and prognostic potential, snoRNAs and SNHGs are being explored as therapeutic targets. However, similar translational hurdles exist. From a therapeutic perspective, snoRNAs and SNHG are of interest as targets for antisense oligonucleotides, small interfering RNAs (siRNAs), and CRISPR/Cas9-based approaches. However, several practical limitations must be considered. Delivery efficiency remains a major challenge, as naked RNA or oligonucleotides are rapidly degraded in vivo. Viral and non-viral vectors are being developed, but each has issues with immunogenicity, off-target effects, and tumour penetration. In the context of bladder cancer, intravesical delivery offers direct access, potentially reducing systemic toxicity; nevertheless, it faces hurdles such as dilution by urine, short retention time, and limited penetration into deeper tumour layers. The stability of RNA-based therapeutics in the urinary environment is also a concern. Currently, all reported targeting strategies for SNHG1, SNHG3, and other SNHGs are at the preclinical stage, and no snoRNA-targeted therapy has entered clinical trials for BC. Therefore, while these molecules are promising targets, extensive optimization and rigorous preclinical testing are required before clinical application.

A specific example of a molecule with therapeutic potential is SCARNA12, which has also demonstrated oncogenic properties in BC. Elevated levels of this scaRNA are associated with extracellular matrix signalling. It has also been shown that silencing this molecule reduces proliferation, migration, invasion, and xenograft growth. This makes SCARNA12 a potential candidate for the development of diagnostic and therapeutic strategies [45].

Taken together, the clinical potential of snoRNAs in BC can be summarized in three main areas: first, early patient risk stratification; second, prognosis of relapse and disease progression; and third, selection of therapeutic targets for molecularly targeted treatment.

Currently, none of these markers have entered routine practice, as most data are obtained from retrospective cohorts and require further validation. Nevertheless, SNORS, SNHG1, SNHG3, and SCARNA12 represent promising candidate biomarkers, though they cannot yet be considered clinically validated.

## 6. Conclusions

This review concludes that small nucleolar RNAs not only participate in the basic process of ribosome biogenesis but are also active, multifunctional regulators involved in key stages of bladder carcinogenesis. Their significant role in complex oncogenic and suppressor signalling pathways is becoming increasingly evident.

In this review, we summarized current data on snoRNA biogenesis and their classical and non-classical functions. Particular attention has been paid to the role of snoRNAs in the development of bladder cancer, a malignant disease with a high risk of recurrence and progression. It has been shown that most of the studied snoRNAs and their host genes act as oncogenes in bladder cancer, and their overexpression correlates with tumour stage, lymph node metastasis and poor prognosis. They stimulate proliferation, epithelial–mesenchymal transition, and invasion, and suppress apoptosis through activation of the PI3K/AKT, Wnt/β-catenin, c-Myc and NF-κB signalling pathways. From a clinical perspective, snoRNAs and the genes that encode them demonstrate high potential as non-invasive diagnostic, prognostic, and therapeutic markers in bladder cancer.

However, a number of key questions remain open. First, most studies have been conducted on limited sample sizes and require validation in independent multicenter cohorts. Second, it remains unclear how snoRNA dysregulation affects their primary function—ribosome biogenesis—and how this relates to non-canonical functions. Future research should aim to decipher tissue- and subtype-specific snoRNA networks and to develop methods for targeting pathological snoRNAs.

## Data Availability

No new data were created or analyzed in this study. Data sharing is not applicable to this article.

## Abbreviations

The following abbreviations are used in this manuscript:

BC	bladder cancer
snoRNA	small nucleolar RNA
SNHG	small nucleolar RNA host gene
sdRNA	snoRNA-derived RNA
miRNA	microRNA
EMT	epithelial–mesenchymal transition
ECM	extracellular matrix
MIBC/NMIBC	muscle-invasive and non-muscle-invasive bladder cancer
SNORS	snoRNA signatures
SNORD/SNORA	C/D box and H/ACA box small nucleolar RNAs
EZH2	enhancer of zeste homolog 2
DNMT3A	DNA-methyltransferase 3A
c-Myc	cellular myelocytomatosis oncogene
